# Assessing Adherence to Antipsychotic Prescribing and Monitoring Guidelines in a Psychiatric Unit for Older Adult Females in Kent and Medway NHS and Social Care Partnership Trust (KMPT): A Retrospective Audit

**DOI:** 10.1192/bjo.2024.605

**Published:** 2024-08-01

**Authors:** Maria Moisan, Bianca Dixon, Ayebatonye Ajiteru

**Affiliations:** Kent and Medway NHS and Social Care Partnership Trust, Dartford, United Kingdom

## Abstract

**Aims:**

Antipsychotic medications are commonly used in the management of psychiatric disorders, including in older adults. However, the use of these medications in older adults can be associated with a higher risk of adverse effects such as cardiovascular event and extrapyramidal symptoms.

This retrospective audit aimed to evaluate adherence to antipsychotic prescribing and monitoring guidelines in a Psychiatric Unit for Older Adult Females in Kent and Medway NHS and Social Care Partnership Trust (KMPT).

**Methods:**

The audit criteria encompassed various aspects of documentation and medication management, including diagnosis documentation, indication, age, comorbidities, consent, baseline assessments, monitoring, review, and follow-up care. Data from two months’ records were analysed leading to an action plan with slight amendments to the user-friendly template for ward round and a physical health monitoring poster for junior doctors and ward staff. These initiatives aim to improve patient care, streamline documentation, while accommodating the rotation of junior doctors. A re-audit is planned post implementation.

This audit’s limitations included the study’s single-site nature, potential sample size constraints and reliance on accurate documentation.

**Results:**

The audit achieved 100% compliance in documenting patient age and MHA status, meeting legal requirements. Weight, BMI, and baseline blood pressure exhibited full compliance. Baseline ECGs had an 86.66% compliance rate, while QTc interval documentation reached 100%. Antipsychotic indication and weekly reviews were documented at 100%, with an 83.33% rate for rationale documentation for medication changes. Comorbidities were fully documented, but extrapyramidal symptom and sedation monitoring showed a 46.66% compliance rate. Baseline blood tests, including glucose, bA1c, lipid profile, electrolytes, renal and liver function, thyroid function, and prolactin levels, generally had high compliance, but lipid profile and liver function achieved 73.33%. Repeat blood tests varied, with electrolytes and renal function at 100%, while thyroid function and prolactin levels scored lower at 26.66% and 46.66%. Continued monitoring of weight, BMI, and blood pressure remained fully compliant. Compliance for repeating ECGs within recommended timeframes reached 53.33%, and recommendations to GPs for yearly ECGs and blood monitoring achieved 50%.

**Conclusion:**

In summary, the audit identified areas of commendable high and medium compliance with antipsychotic prescribing guidelines in a Psychiatric Unit for Older Adult Females in KMPT. An action plan has been formulated to not only enhance patient care but also to refine the documentation process positively further, fostering continued progress in the provision of high-quality care.

